# Autonomic Neuropathy in Diabetes Mellitus and Obesity: An Update

**DOI:** 10.1155/2011/607309

**Published:** 2011-12-01

**Authors:** Nicholas L. Katsilambros, Andrew J. Boulton, Nicholas Tentolouris, Alexander Kokkinos, Stavros Liatis

**Affiliations:** ^1^First Department of Propaedeutic Medicine, University of Athens Medical School, Laiko General Hospital, 17 Ag. Thoma Street, 11527 Athens, Greece; ^2^Research Laboratory “Christeas Hall” and Evgenideion Hospital, Athens University Medical School, 5 Doryleou Street, 11521 Athens, Greece; ^3^Department of Medicine, University of Manchester, Manchester Royal Infirmary, Oxford Road, Manchester M13 9WL, UK

Autonomic neuropathy, although not rare, is one of the most insidious complications of diabetes mellitus, especially in those patients with long-standing and poorly controlled disease. A lot of attention has been given to the cardiovascular aspect of autonomic dysfunction, which has been implicated in increased mortality, especially in view of recent reports regarding the association of very tight glycaemic control with increased mortality, which could be attributed to hypoglycemia-induced arrhythmias.

However, cardiac autonomic neuropathy is only one of the many facets of this complication, which leaves virtually no organ system unaffected. In this special issue of the journal, we have invited authors to submit reviews and research articles regarding autonomic neuropathy in patients with diabetes and obesity, since this last condition is also associated with autonomic dysfunction, due to concomitant insulin resistance. The issue includes papers on cardiac autonomic neuropathy, sudomotor dysfunction, skin blood perfusion, insulin secretion, and exocrine pancreatic insufficiency.

In an extensive and elaborate review, Dr. C. Voulgari et al. examine the application of a simple, inexpensive, yet extremely useful tool, the electrocardiogram, in assessing diabetic patients with possible cardiac autonomic neuropathy and screening them for cardiovascular disease.

In the following research papers, H.-W. Huang et al. investigate the applicability of using frequency domain analysis on laser Doppler flowmetry data in the study of carpal tunnel syndrome and diabetic polyneuropathy, while N. Papanas et al. examine the association between serum uric acid levels and sudomotor dysfunction in patients with type 2 diabetes mellitus and find it to be positive, a fact which leads them to speculation on the potential role of serum uric acid in sudomotor dysfunction.

Returning to cardiac autonomic function, Dr. S. Liatis et al. demonstrate that in young patients with type 1 diabetes, even in the absence of macrovascular and renal complications, large arterial stiffness can be present and diagnosed with pulse wave velocity analysis, and, furthermore, cardiac parasympathetic function, as assessed by the classic tests proposed by Ewing and Clarke, is strongly associated with this condition, thus highlighting the importance of cardiac autonomic dysfunction in predicting cardiovascular disease risk, even in the absence of overt diabetic complications.

Apart from diabetes, obesity, a condition which predisposes to diabetes and is characterized by insulin resistance, has been proposed to be associated with impaired autonomic function. This is addressed in the research paper by Dr. A. E. Andreazzi et al., in which prediabetic obese rats are demonstrated to have low sympathoadrenal activity, which, according to the authors, contributes to glucose intolerance and hyperinsulinemia during an intravenous glucose tolerance test.

Finally, Drs. P. D. Hardt and N. Ewald present an elegant review on exocrine pancreatic insufficiency, in which they address its increased frequency in type 1 and 2 diabetes and discuss whether it is a complication of the disease or a concomitant condition to underlying pancreatic diseases which also lead to diabetes, and the possible role of diabetic neuropathy in its manifestation, after carefully guiding the reader through the relevant literature.

The present special issue should by no means be considered a comprehensive “primer” on all the facets of autonomic neuropathy, and this was never its original aim. The purpose was to present the reader with an update on as many as possible of the aspects of autonomic dysfunction in diabetes and obesity, and in these terms, we consider it to have achieved its goal. We believe that both the research papers, as well as the review articles contained herein are of high quality and address key issues in the presentation and diagnosis of this condition. We hope that this issue will underscore the importance of autonomic dysfunction as a condition that is not at all rare, but is often overlooked by clinicians both in terms of diagnosis as well as treatment and that it will also serve as a reminder of the need for further research in this field.

*Nicholas L. Katsilambros* *Nicholas L. Katsilambros* *Andrew J. Boulton* *Andrew J. Boulton* *Nicholas Tentolouris* *Nicholas Tentolouris* *Alexander Kokkinos* *Alexander Kokkinos* *Stavros Liatis* *Stavros Liatis*

